# Room-Temperature Framework Oxygen Isotope Exchange during Interaction of Water with Hydrophilic Pure-Silica Zeolites Studied Using Nuclear Magnetic resonance Spectroscopy and Neutron Diffraction

**DOI:** 10.1021/jacs.5c23024

**Published:** 2026-03-17

**Authors:** Nicole L. Kelly, Maximillian G. Stanzione, Deborah Brako-Amoafo, Gaynor B. Lawrence, Cameron M. Rice, Paul S. Wheatley, Jonathan M. Keys, Christopher J. Heard, Henry E. Fischer, Alexandra S. Gibbs, Sharon E. Ashbrook, Russell E. Morris

**Affiliations:** † EaStCHEM School of Chemistry and Centre for Magnetic Resonance, 7486 University of St Andrews, St Andrews, KY16 9ST U.K.; ‡ Department of Physical and Macromolecular Chemistry, Charles University, Hlavova 8, Prague 2 12800, Czech Republic; § Institut Laue-Langevin, 6 Rue Jules Horowitz B.P. 156, Grenoble, Cedex 9 38042, France

## Abstract

Four different hydrophobic, low-defect pure-silica zeolites are contacted with isotopically enriched water at room temperature, and all show isotope exchange of ^17^O into the Si–O–Si units of the zeolite structure using multiple-quantum MAS NMR spectroscopy. For the CHA zeolite structure, exchange of ^18^O into the framework was identified using neutron diffraction—the first time this technique has been used to differentiate oxygen isotopes in any solid-state material. The results indicate that even supposedly very hydrophobic pure-silica zeolites are susceptible to oxygen exchange under very mild aqueous conditions. This has important consequences for our view of the structure of zeolites in contact with water.

## Graphical abstract

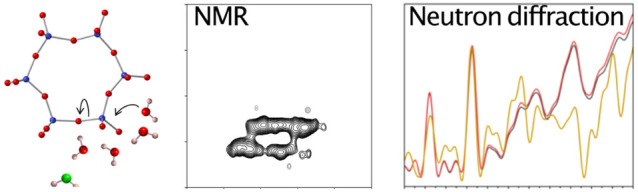

## Introduction

Zeolites are an industrially important family of microporous oxides, with applications in several different fields. Ever since the first publication of their synthesis, pure-silica zeolites have been noted for their hydrophobic properties.^1^ This feature has led to intriguing recent potential applications of hydrophobic zeolites as O_2_ carriers in simulated physiological solutions—a type of artificial blood^2^—and as the O_2_ source for electrocatalytic reactions.^3^ The use of hydrophobic zeolites has also been postulated for important separations, such as alkenes from alkanes.^4,5^ These applications rely on the selectivity induced by the pore openings into the zeolitic structures.^4^ The generally accepted, textbook view of zeolites is that their pores do not change markedly except for thermal vibrations. In addition to the different applications, the field of pure-silica zeolites has been given a recent boost by the development of nontraditional synthetic protocols that rely on the topotactic condensation of silica layers^6–8^ and chains^9–12^ into three-dimensional materials.

Experimental and computational studies show that intrusion of water into the hydrophobic pores of pure-silica zeolites occurs only at high pressures in the region of MPa.^13,14^ The elegant density studies reported by Mason,^3^ indicate that negligible amounts of water are incorporated into the pores of the zeolite, even when the material is dispersed in aqueous liquid. In contrast to this expectation of limited interaction with moisture, here we show that contacting liquid ^17^O- or ^18^O-enriched water at room temperature with pure-silica zeolites leads to enrichment of the framework Si–O–Si units as proved by solid-state NMR spectroscopy and neutron diffraction methods. We show that the enrichment occurs surprisingly quickly (within a few hours) even at room temperature. The level of enrichment is well above that which would be expected if the exchange occurred only at defect sites or at the surface of the crystallites.

Neutron diffraction is a powerful technique for determining structure and can discriminate between isotopes as the scattering depends on nuclear structure. It is particularly useful for experiments where there is a large difference in scattering cross section between the isotopes, primarily those of hydrogen. However, there have been no previous neutron diffraction experiments on solids that have discriminated between oxygen isotopes because of the small difference in scattering amplitude of only about 3%. Here we also show how neutron diffraction can be used to confirm the isotopic exchange in the zeolite framework.

Enrichment in ^17^O/^18^O at the levels measured indicates that in the presence of water even at room temperature the framework Si–O–Si bonds must be constantly breaking and reforming. This has consequences for our fundamental view of zeolites as materials with cavities and pores that do not change with time, and in particular for how we view the selectivity of zeolites in aqueous conditions, which will become more important as we transition away from petroleum-based processes toward less carbon-intensive (e.g., biorefinery) technologies.^15^ It will also be important for our understanding of developing technologies such as microporous water^2^ that rely on hydrophobicity of the zeolites for their utility.

## Results

Full materials and methods for the studies can be found in the Supporting Information. The study focused on four different zeolite frameworks with the AFI, CHA, FER, and MFI topologies.^16^ The pure-silica zeolites were prepared using adapted recipes taken from the literature and were calcined at high temperature to remove any organic structure-directing agents (OSDAs).^1,17–19^ The topology and phase purity of the zeolites were confirmed using powder X-ray diffraction (PXRD) and ^29^Si MAS NMR experiments were used to assess the defect levels in the materials by assessing the number of Q^3^ silanol species ((SiO_3_)-Si–OH) present compared to the fully connected Q^4^ tetrahedral species. Only in the case of zeolite CHA was it possible to measure the level of Q^3^ silanols using single-pulse ^29^Si MAS NMR measurements. In the case of zeolites AFI, MFI, and FER there was no discernible signal in either the ^29^Si MAS or the ^1^H/^29^Si cross-polarization (CP) MAS NMR spectra, indicating a very low level of silanol defects (see Supporting Information Section S1.3 for full details).

Interaction with water at room temperature was completed in two main ways: Method 1, “slurrying”, involved contacting enriched water (40% H_2_^17^O) with the zeolite inside a PTFE HRMAS insert while method 2 involved contacting the zeolite with a larger volume of enriched water while either being stirred or shaken. In the latter two cases, after 7 days contact with water the sample was filtered and dried at 80 °C. The advantage of the former method is that the same material can be studied over time (up to months) but the ^17^O NMR single-pulse spectra can be dominated by water and so difficult to analyze accurately. Another potential drawback of the slurrying method is that fast spinning during the reaction and data collection itself may induce intrusion of water into the zeolites because of centrifugal forces. The advantage of method 2 is that the sharp, high intensity signal from free water is removed by the drying step, allowing easier quantification of the enrichment level by comparison of single-pulse (short flip angle) MAS NMR intensities with the spectrum of a reference with known ^17^O content (see Supporting Information, Section S1.3). The drying step after stirring or shaking also means that there is no free water present while the sample is spun at high rates during NMR data collection. This means any chance of MAS-induced water intrusion into the zeolites is reduced using this method.

### Zeolite AFI—Solid-State NMR Spectroscopy

Figure 1 shows the results of contacting pure-silica zeolite AFI with water using method 1 (slurrying). Immediately after initial contact with water, the sample was sealed inside the rotor and NMR spectra acquired. The single-pulse one-dimensional ^17^O MAS NMR spectra (Figure 1c) are dominated by the presence of the liquid water, but the ^17^O triple-quantum MAS NMR spectra, which do not contain any signal from dynamic water, show clear evidence of signal from the Si–^17^O–Si oxygen species, at δ_1_ of ∼30 ppm (consistent with literature^20–24^ ). The signal develops in intensity to approximately 7 days, after which the intensity of the signal does not then change appreciably with time (Figure S2). After 212 days in contact with water the sample was recovered from the rotor and dried at 80 °C. ^29^Si MAS NMR experiments (Figure 1b) and powder X-ray diffraction (Figure S3) indicated that the sample was still highly crystalline, with no evidence of any degradation. These data show that there is a clear and rapid substitution (at least on the time scale of collecting an NMR spectrum) of ^17^O for ^16^O into the Si–O–Si bonds of the framework. Such rapid enrichment of the framework is surprising in itself and raises several questions, including whether zeolite AFI is a special case or whether the observation is true for other pure-silica zeolite frameworks.

**Figure 1:**
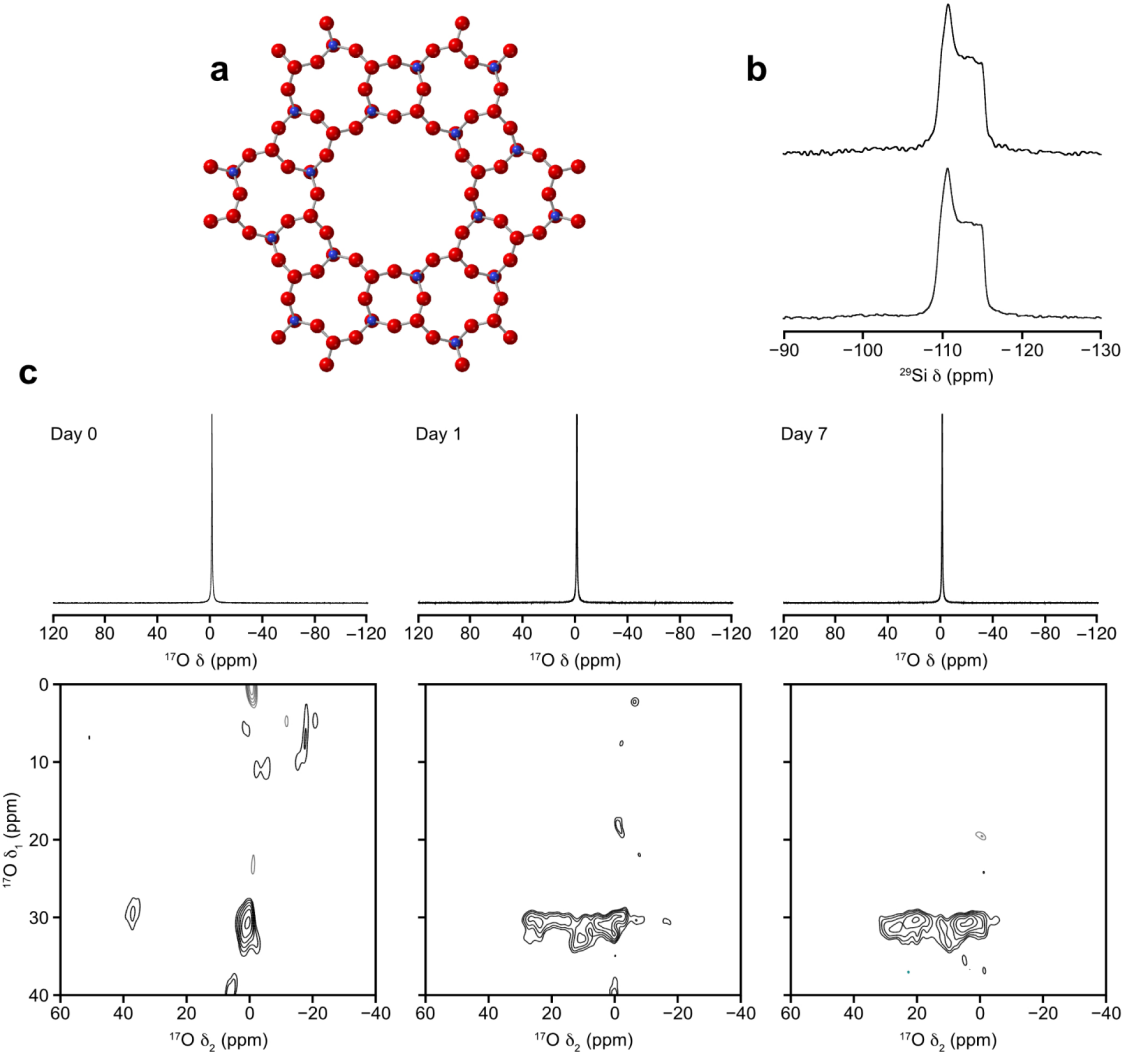
Pure-silica zeolite AFI in contact with ^17^O-enriched water. (a) The idealized framework structure of zeolite AFI. Key: silicon = blue, oxygen = red. (b) Single-pulse ^29^Si MAS NMR spectra (9.4 T, 10 kHz) of calcined zeolite AFI before (bottom) and after (top) contact with ^17^O-enriched water using the slurrying method. (c) ^17^O (14.1 T, 20 kHz) single-pulse ^17^O MAS NMR (top) and triple-quantum MAS NMR (bottom) spectra of calcined zeolite AFI from left to right immediately after contact with ^17^O-enriched water, after 1 day and after 7 days.

### Zeolite CHA—Solid-State NMR Spectroscopy

The structure (Figure 2a) of pure-silica CHA is not as complex as other zeolites (especially AFI, which is disordered), with only one crystallographically distinct silicon site and four oxygen sites, which makes it very suitable for detailed structural studies. Single-pulse ^29^Si MAS NMR spectra show only one signal for Q^4^ silicon species and a small signal indicating the presence of Q^3^ defects comprising ∼3% of the total silicon atoms (Figure S1).

**Figure 2:**
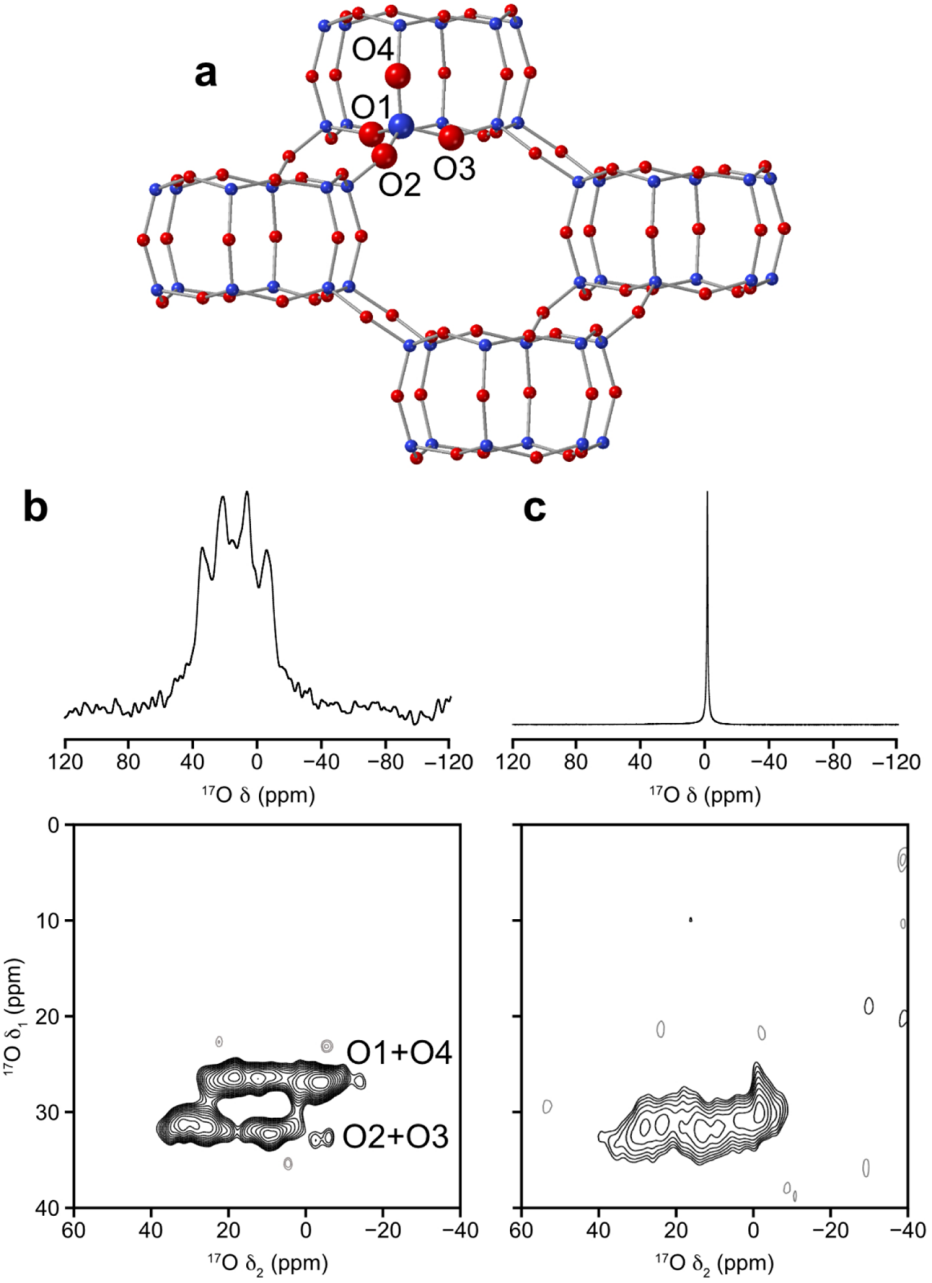
^17^O enrichment in pure-silica zeolite CHA. (a) The crystal structure of zeolite CHA showing the numbering system for the atoms used in this study. Key: silicon = blue, oxygen = red. ^17^O (14.1 T, 20 kHz) single-pulse ^17^O MAS NMR (top) and triple-quantum MAS NMR (bottom) spectra, of (b) calcined ^17^O-enriched CHA (synthesized with 40% enriched water) with the assignment of the resonances marked; and the same spectra for (c) CHA (synthesized with natural abundance water) after slurrying with ^17^O-enriched water for 7 days.

Slurrying of CHA with ^17^O-enriched water in the same way as described above for AFI showed a very similar result. After 7 days in contact with water at room temperature the ^17^O triple-quantum MAS NMR spectrum showed clear evidence of substitution of ^17^O for ^16^O in the Si–O–Si bonds. PXRD of the recovered solid at the end of the experiment showed no degradation of the crystalline structure of the framework but the ^29^Si MAS NMR spectra showed an increase in the number of Q^3^ defect sites (up to ∼9%) in the material. To quantify the level of enrichment, method 2 was used as described above. The single-pulse (short flip angle) ^17^O MAS NMR spectrum was used to calculate the enrichment level by carefully comparing the intensity of the signal with a known standard (see Methods section in the Supporting Information), giving a measured enrichment in ^17^O of ∼17% for the stirred CHA sample.

One should remember that the water used in the exchange reaction is only enriched to 40% in ^17^O. If we assume the level of enrichment will scale linearly with % enrichment of the water, this is equivalent to an enrichment in Si–O–Si of ∼42% if 100% ^17^O-enriched water were to be used. This is important in comparing the enrichment level obtained from the neutron diffraction studies when using 98% ^18^O-enriched water (see below). For the micron-sized, cuboid crystals we have in the CHA sample (Figure S6), the ratio of surface silanols to bulk silicon is approximately 0.002, and so we can rule out any explanation that relies on interaction only at the surface of the material. The water must therefore be penetrating into the crystallites.

The only major difference between the slurried and stirred samples (methods 1 and 2) is that the number of defects in the sample after stirring is significantly higher (17%) than after slurrying (9%). Scanning electron microscope images showed that after stirring the external surfaces of the CHA crystals were roughened, while there was no such effect after slurrying (Figure S6). A less energetic agitation technique (shaking) was also used, and this reduced both the surface roughening of the crystals (Figure S6) and the number of Q^3^ defects measured in the ^29^Si NMR spectrum (to ∼5%). However, this also reduced the level of ^17^O enrichment of the framework from ∼17% for the stirred sample to ∼9% for the shaken sample.

In all cases the level of enrichment in the zeolite is well above the initial level of defects. However, this does not mean that defects are not important in the enrichment process. The presence of Q^3^ silanols will naturally act as a nucleation point for the small clusters of water that both modeling and neutron diffraction experiments (see below) suggest are present in the materials. Defects are therefore likely to be important as the sites for water clustering.^13^

As a reference, a sample of CHA was prepared where ^17^O-enriched water was added directly to the hydrothermal synthesis. The measured ^17^O enrichment level for the sample enriched during synthesis was ∼38%. This is relatively close to the maximum possible level of ∼40%. Interestingly, the ^17^O triple-quantum MQMAS spectrum of this sample (Figure 2c, Figure S7) showed a significant difference to those observed from postsynthetic enrichment. The as-synthesized ^17^O CHA had two clear signals in the spectrum, while those from the postsynthetic enrichment showed only one broader signal. This suggests that there may be some selectivity in the postsynthetic enrichment process. To further understand the NMR spectra, first-principles density functional theory (DFT) calculations were used to assign the signals as shown in Figure 2b, and full details of the calculated NMR parameters are given in Section S1.5 and Table S3 in the Supporting Information. There is a broadening and small shift of the ^17^O MQMAS signals in the δ_1_ dimension for all samples that have been post synthetically treated with water (whether stirred, shaken or slurried) and this makes assignment of the resonances less definitive than one would hope, as we cannot rule out the possibility that all four ^17^O sites are now contained in the one broader signal. The broadening of the signals is likely due to varying local environments caused by the small amounts of water that are present within the pores, and it is not surprising that the position of the signals in the δ_1_ dimension varies slightly between enrichment methods (Figure S7).

### Zeolite CHA—Computer Modeling

The NMR studies suggest that the ^17^O enrichment in pure-silica CHA could be regioselective (or at least preferred) at the O1 or O4 sites, although given the broadening of the signal in the 3Q MAS NMR spectra (see above) this is only a suggestion. To probe this possibility, we used computational methods to calculate the relative electronic energies of materials with silanols formed at each of the four possible oxygen sites. Whatever the precise mechanism of the oxygen exchange process (see below for discussion) the first step must be the breaking of an Si–O–Si bond by addition of ^17^O-enriched water to form silanol species, which will then react further to exchange and then reform the Si–^17^O-Si bonds. Reactive neural network potentials (NNP) were employed in molecular dynamics simulations (NNP-MD, 1 ns time scale with a time step of 0.5 fs). Simulations were performed with both defect-free (i.e., all Q^4^) framework models and those with silanols formed by breaking the Si–O–Si units at each of the oxygen sites. Two water loadings were considered: anhydrous CHA (0 water molecules per cell), and water at a density similar to that seen liquid water (corresponding to 14 water molecules per unit cell). The results indicate that silanol sites formed by breaking Si–O1–Si and Si–O4–Si are of lower energy than silanols formed when the Si–O2–Si and Si–O3–Si bonds are broken (Table S1, Figure S9). At first sight, these results support the tentative conclusion that the single ridge in the ^17^O MQMAS NMR spectra (Figure 2) may come from regioselective exchange at only certain sites in the structure. However, a back exchange experiment, taking a material enriched in ^17^O during synthesis followed by stirring for 7 days in natural abundance water, showed a reduction in intensity of both the ridges in the ^17^O 3QMAS NMR spectra. If there was selective enrichment we would expect one of the ridges in the spectra would considerably reduce in intensity more than the other. There is no obvious or major difference in the reduction of intensity of the two signals and the spectra show a similar increase in broadening to other samples that have been water treated (Figure S8). We can therefore conclude that the difference in energetics of the different silanol species from the computational studies is not enough to manifest itself as clear and obvious regioselectivity in the isotopic enrichment. The optimized atomic positions for water in the pores were then used as the starting model for subsequent neutron diffraction studies.

### Zeolite CHA—Neutron Diffraction

Further confirmation of the oxygen exchange in the silica CHA zeolite is provided by neutron diffraction experiments on ^18^O-enriched materials prepared using the stirring method with 98%-enriched water. One might imagine that for relatively well-crystallized materials such as zeolites, Rietveld refinement of models against powder diffraction data would be a good approach.^25^ Unfortunately, the well-known correlation between atomic displacement parameters and scattering factors makes this difficult for such small differences in isotope scattering lengths.^26^ An approach known as Neutron Diffraction with Isotope Substitution (NDIS)^27^ is used instead. The method relies on accurate subtraction of the diffraction intensities from two chemically and structurally identical samples: one containing a natural abundance of oxygen isotopes, and one that has been enriched in ^18^O. The resultant Q-space function is called the First-Order Difference (FOD) and can be Fourier transformed give the FOD-PDF, a real-space function that exhibits peaks only at internuclear distances containing exchanged ^18^O, and with amplitudes proportional to the fraction of exchanged oxygen atoms.

Accurate NDIS experiments require precise knowledge of neutron scattering lengths for the isotopes in question, which in the case of ^18^O was recently determined to be b_coh_(^18^O) = 6.009(5) fm,^28^ as compared to that for the naturally occurring isotopic composition, b_coh_(^nat^O)= 5.805(4) fm, giving a scattering length contrast of 0.204(3) fm. We postulate that even this very small difference is sufficient for modern neutron diffraction instrumentation to differentiate.^28,29^ Note that this entirely empirical PDF analysis method is free from any modeling bias.

In Figure 3a, a comparison of the radial distribution function (RDF), directly derived from the PDF, for ^nat^O-CHA and ^18^O-enriched CHA with the FOD-RDF (gold curve) shows clear peaks at the expected Si–O and O–O distances. Even with such small differences in scattering the FOD-RDF shows extremely clear evidence of enrichment. To quantify the enrichment, accurate normalization of the diffraction intensities is required, after taking into account sample attenuation corrections and the subtraction of low and stable instrument backgrounds. By adjusting the average ^18^O-enrichment level in the data normalization, the 2.62 Å peaks of all three curves can be brought into correspondence. This results in an estimated ^18^O enrichment of ∼50%, with an estimated uncertainty of ± 5% (see Section S1.4 for discussion of uncertainties in the experiment). In addition, this results in near zero amplitude of the FOD curve at 5.35 Å, which should be the case since there are no interatomic distances at 5.35 Å in the CHA structure involving oxygen atoms. The level of oxygen exchange estimated from the neutron diffraction (50%) is slightly more than that from the NMR measurements (estimated at 42% for the CHA treated in the same way—see above). However, one should note that the sharpness of the peaks in Figure 3a gives rise to some unavoidable Fourier “ripples” around each peak, and such artifacts are naturally exaggerated in the experimental FOD that results from a small diffraction signal. These issues limit the uncertainty on the estimated enrichment to approximately ± 5% (see Section S1.4). Given the uncertainty on the NMR experiments is also of the order of 3–5%, we cannot conclusively state that the two results are different.

**Figure 3:**
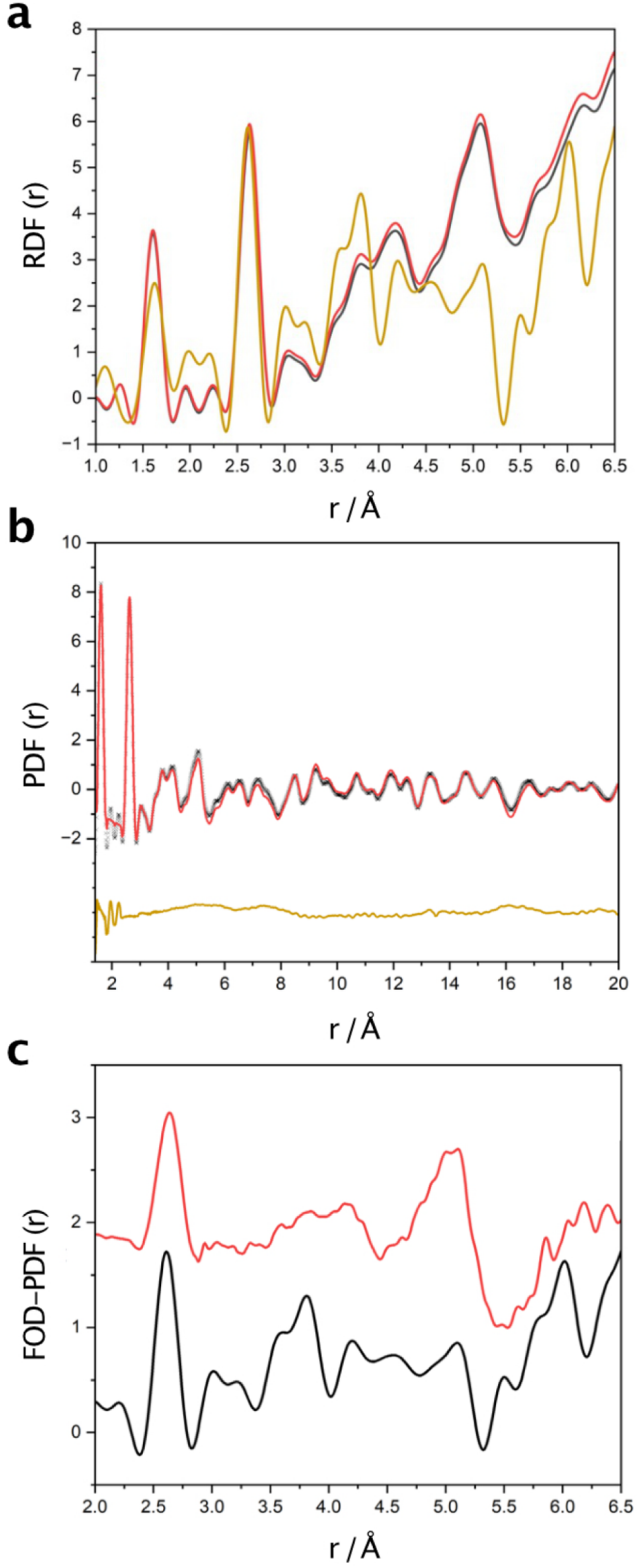
Neutron diffraction studies on isotopically enriched zeolite CHA. (a) The Fourier transforms of the total diffraction patterns for the ^nat^O CHA zeolite (black curve) and ^18^O-enriched CHA zeolite (red) in units of Å^–1^ atom^–1^, as well as for their FOD-RDF (gold). The FOD-RDF has been scaled to match the intensity of the peak at 2.62 Å, which is due solely to O–O interatomic distances (peaks for the Si–O and O–O interatomic distances can be unambiguously assigned and are labeled). (b) The fit of the hydrated zeolite model to the neutron PDF­(*r*) data (units: Å^–2^ atom^–1^) for the ^18^O-enriched CHA. Note the difference curve is offset from zero for clarity (black = experimental data, red = calculated and gold = difference curve). (c) A comparison of the calculated FOD-PDF (red) and the experimental FOD-PDF (black). Note that the main peaks in the experimental FOD-PDF are reproduced by the calculation but not the artifacts caused by the Fourier “ripples” in the measurement.

Figure 3b shows the results of a refinement of a structural model for ^18^O-enriched CHA against the PDF­(*r*), formerly known as the density function, *D*(*r*), with a similar refinement performed for the ^nat^O CHA sample. The initial structural models contained only the atoms in the zeolite framework and refined to give *R*_w_ values of 24.1% and 20.3% for the ^nat^O CHA and ^18^O CHA samples, respectively. However, including a small amount of extraframework water within the pores of the zeolite, with starting positions taken from the computational results above, resulted in an improved fit between experimental and calculated PDF­(*r*) functions and *R*_w_ values of 17.7% and 17.8% for the ^nat^O CHA and ^18^O CHA samples, respectively. Details of the PDF refinements can be found in the Supporting Information. These results allow us to calculate a FOD-PDF and compare to the experimental version (Figure 3c). The main features in the calculated FOD-PDF, and especially the peak due to the O–O distances at ∼2.6 Å, reproduce those of the experiment well, giving confidence in the applicability of the basic assumption that the only difference between structures is the isotopic labeling. However, the measured FOD results from very small differences in scattering amplitude and it is therefore not surprising there is some mismatch.

### Zeolites FER and MFI

Two additional pure-silica materials with the FER and MFI topologies were prepared using methods that are known to produce very low defect (sometimes called defect-free) zeolites.^30^ Indeed, the level of defects were below the levels of quantification in the NMR experiments for both materials. Figure 4 shows that the Si–O–Si oxygen species were enriched in ^17^O as was seen for the other pure-silica zeolites, with only small changes in the numbers of defects and crystallinity (see also PXRD data, Figures S11 and S12). The FER sample did show an additional low intensity peak in the PXRD pattern indicating that a small amount of framework degradation occurred even at room temperature (Figure S11). All four of the pure-silica zeolites tested in this work showed similar lability toward oxygen exchange in the Si–O–Si units and so we believe that the findings are likely to be general for all pure-silica zeolites. However, there is a difference in the absolute enrichment levels depending on the topology. For FER, the measured level of enrichment after stirring for 7 days in 40% ^17^O-enriched water was only 4%, while for MFI the enrichment level was 14%. We can say that CHA (measured enrichment level 17%) and MFI are more susceptible to this room temperature isotopic enrichment than FER.

**Figure 4:**
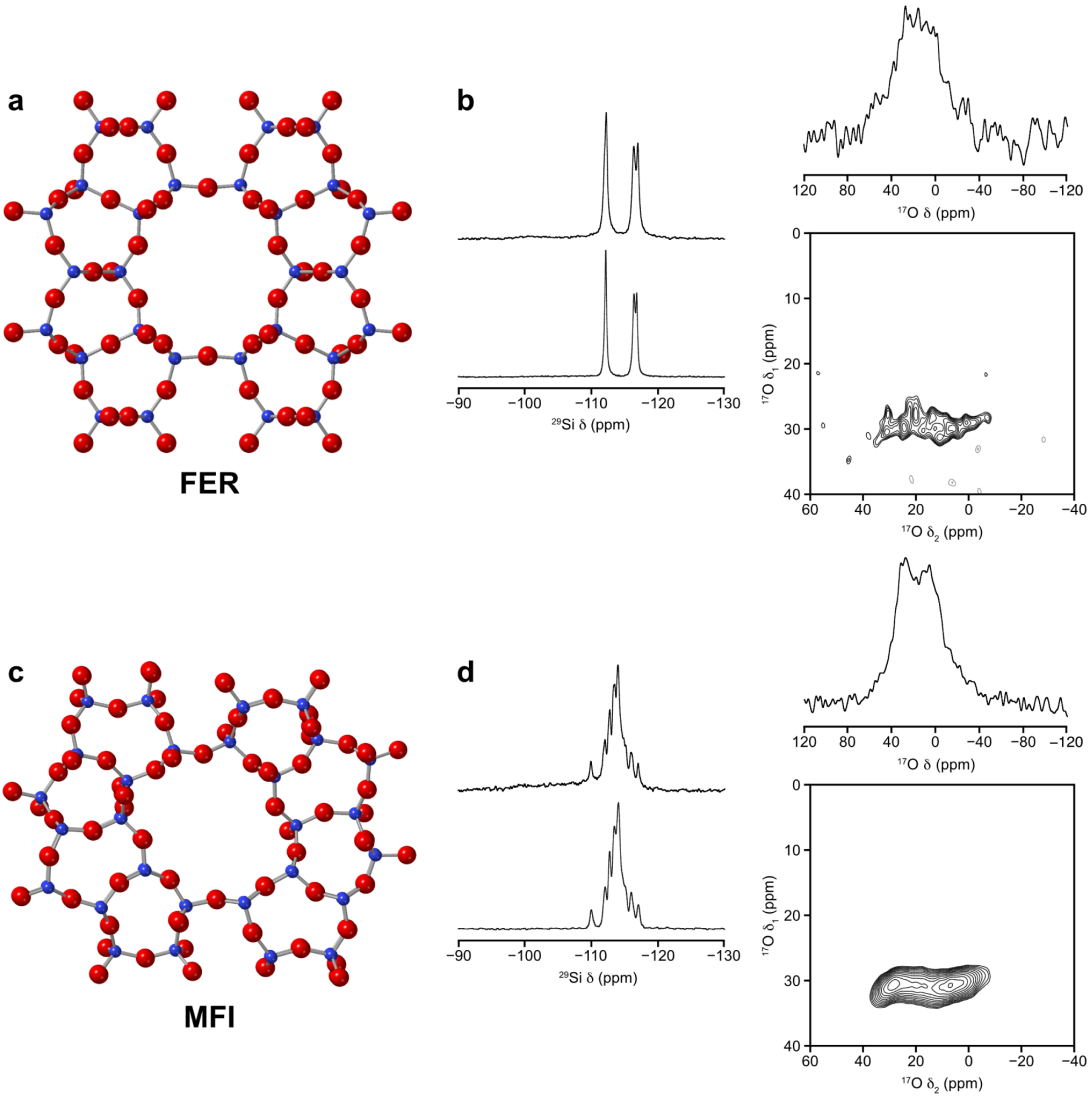
Oxygen isotope exchange in pure silica zeolites FER and MFI. (a and c) The crystal structures of zeolite FER and MFI respectively; (b and d) Single-pulse ^29^Si MAS NMR spectra (9.4 T, 10 kHz) of calcined zeolite (b = FER, d = MFI) before (bottom) and after contact with enriched water and stirring for 7 days (top). Also shown for each sample are the single-pulse ^17^O MAS NMR and ^17^O triple-quantum MAS NMR spectra (14.1 T, 20 kHz) of the enriched material. Key: silicon = blue, oxygen = red.

## Discussion

Oxygen exchange into the framework is relatively facile at room temperature for pure-silica zeolites AFI, CHA, FER, and MFI with enrichment levels well above the number of the defects present in the materials. While this is known for hydrophilic zeolites where water is expected to interact strongly with the framework,^20–24,31,32^ this is a significant surprise given the often-stated hydrophobic nature of pure-silica solids, and the results have significant consequences for how we view the structure of such zeolites. The multiple bond-breaking and remaking required for isotopic substitution, such as that shown in Figure 5a, means that the integrity of the pore windows will not be maintained when in contact with aqueous systems. This, in turn, will significantly affect which molecules/ions will have access to the internal surface at any one time—the basis of shape/size selectivity in zeolites. This contrasts with the textbook picture of zeolites with consistent pore windows (shown in Figure 5b as the structure inside the blue box). This inevitably means that selectivity for separations, for example, in aqueous systems will necessarily be more complex than that in anhydrous situations where the pore windows of the zeolite will likely remain intact.

**Figure 5:**
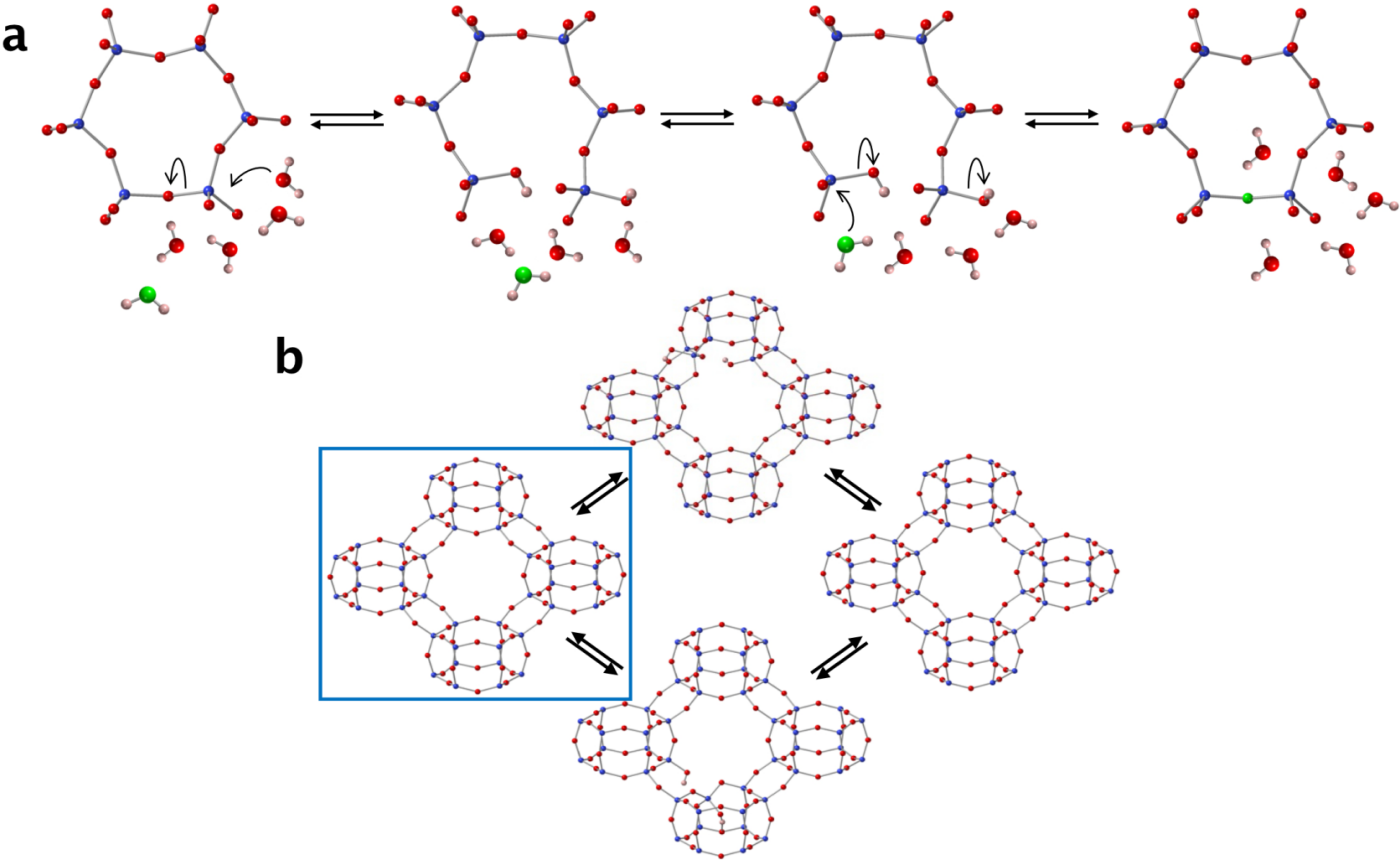
Possible mechanism for oxygen isotope exchange in pure silica zeolites. (a) shows a possible mechanism for water-induced Si–O–Si bond cleavage in aluminosilicate zeolites. Key: silicon = blue, oxygen = red, hydrogen = pink. The ^17^O/^18^O atom that ends up in the framework is shown in green. Note that this process requires several water molecules to shuttle protons to and from the silanols as Si–O–Si bonds are broken and reformed. (b) The textbook view of zeolites is as a set of pores where the bonds remain intact at all times (shown inside the blue square). However, the mechanism shown in (a) requires multiple bonds to be continually broken and remade in the presence of water. The integrity of the pore windows must be transient so that the instantaneous structure will not necessarily have fully formed windows at any one time.

Of equal importance, and perhaps of even more general significance, is proof that neutron diffraction can be used to characterize isotopic differences in oxygen between samples. Given the small difference in scattering power between ^16^O and ^18^O such clear confirmatory evidence provided by the FOD approach we describe here may come as a significant surprise to some researchers in the area. Given the overwhelming importance of oxide materials in many different technologies, the demonstration that neutron diffraction can be used to understand isotopic substitution adds another weapon in our armory of how to characterize these important solids.

## Supplementary Material



## Data Availability

The NMR research data supporting this publication can be accessed at https://doi.org/10.17630/402d2f05-503f-4ae1-a077-38373caf5f9c. The neutron diffraction data can be accessed at doi: 10.5291/ILL-DATA.6-06-526.

## Notes

Experimental details, materials and methods, supplementary figures featuring further results, and tables of computed data (PDF)
