# Supplementary material for: Room-Temperature Framework Oxygen Isotope Exchange during Interaction of Water with Hydrophilic Pure-Silica Zeolites Studied Using Nuclear Magnetic resonance Spectroscopy and Neutron Diffraction
Source: J Am Chem Soc. 2026 Mar 17;148(12):13257–65. doi: 10.1021/jacs.5c23024 (PMC13047528; doi:10.1021/jacs.5c23024)
Supplement: Supplementary file 1 [file ja5c23024_si_001.pdf]

Room temperature framework oxygen isotope exchange on interaction of water with hydrophobic pure-silica zeolites studied using nuclear magnetic resonance spectroscopy and neutron diffraction

Nicole L. Kelly,[1] Maximillian G. Stanzione,[1] Deborah Brako-Amoafo,[1,2] Gaynor B. Lawrence,[1] Cameron M. Rice,[1] Paul S. Wheatley,[1] Jonathan M. Keys,[1] Christopher J. Heard,[2] Henry E. Fischer,[3] Alexandra S. Gibbs,[1] Sharon E. Ashbrook,[1*] Russell E. Morris[1*]

Corresponding authors: sema@st-andrews.ac.uk, rem1@st-andrews.ac.uk

The file includes:

1. Materials and Methods, including synthesis of samples, experimental procedures for isotopic enrichment, experimental details for NMR spectroscopy and neutron diffraction analysis, and computational details.
2. Supplementary Figures S1 to S12 showing further results.
3. Tables S1, S2 and S3 with results of computational work.

S1 Materials and Methods

S1.1 Synthesis of Zeolites

The pure silica zeolites were prepared using adapted recipes taken from the literature and were calcined at high temperature to remove any organic structure-directing agents (OSDAs) (See references in main text). ^{29}Si MAS NMR (Fig. S1) experiments were used to assess the number of silanol defects by measuring the amount of Q^3 silica species ((SiO_3)-Si-OH) present compared to the fully connected Q^4 tetrahedral species. The topology and phase purity of the zeolites was confirmed using powder X-ray diffraction (PXRD), see Figs. S3, S5 and S10, and scanning electron microscopy (SEM) was used to monitor crystal morphology (Figs. S4 and S6).

Chabazite synthesis

For the neutron diffraction experiments the following synthesis was used to prepare chabazite samples.

OSDA preparation

0.09 mol, 13.709 g of adamantylamine and 0.225 mol, 32.067 g of potassium carbonate were added to a round bottomed flask with 120 mL dimethylformamide (DMF). This solution was stirred in an ice bath, covered with foil, whilst 0.2726 mol, 38.7 g of methyliodide was added dropwise over 30 minutes. Once all the methyliodide was added, the ice bath was removed and the reaction was heated to 50 °C under reflux for 10 days, leaving the foil covering.

After 10 days the potassium carbonate was filtered off, the resultant solution added to 120 mL more DMF and refluxed at 50 °C for four hours to help gather more product. Once all the filtrate was gathered, ethyl acetate was added to precipitate out the iodide product. The filtered product was then recrystallised in a small volume of water. 6.4 g of the product was dissolved in deionised water passed over an ion exchange resin, which was prewashed twice for 30 minutes, to provide the OSDA with a hydroxide anion. The ion exchange was performed twice for around 3 hours each time. The solution was concentrated to ~0.5 M using the rotary evaporator. Verification of the OSDA:OH ratio was carried out through an imidazole test using NMR spectroscopy to compare the experimental ^1H NMR spectrum against the calculated concentration. This was performed by dissolving the calculated quantity of 2-methyl imidazole in the OSDA.OH solution and checking the relative intensity of CH_3 peaks in liquid-state ^1H NMR spectra.

CHA preparation

25 mL of 0.54 M OSDA solution (0.0135 mol) was added to a Teflon cup with 3.75 g, 0.018 mol, of tetraethyl orthosilicate (TEOS). This solution was stirred to hydrolyse overnight (this will dry significantly), then 489 μL of HF was added to the stirring solution and it was left stirring again for a further two days. After this was completed, the vessel with stirrer was weighed to determine if the desired synthesis ratio of 1 SiO_2 : 0.75 SDA.OH : 0.75 HF : 5 H_2O had been reached. If the mass was too high, then the reaction was left to stir once more, and if too low the correct weight of distilled water was then added. For the case of the CHA sample prepared with ^{17}O-enriched water in the reaction mix, the solution was stirred for a longer time until the ratio of H_2O : SiO_2 was lower than 5 : 1. At this point 40% ^{17}O-enriched water was added (~0.5 mL) to return the synthesis ratio back to 1 SiO_2 : 0.75 SDA.OH : 0.75 HF : 5 H_2O.

The stirred product was then split between two Teflon-lined autoclaves and placed in a rotating oven. The autoclaves were then heated to 150 °C and then held for 12 hours without the rotation turned on. At this point the sample was held at 150 °C while rotating at 10 rpm for 168 hours, before cooling to 20 °C at 150 °C/hour. The sample was then collected through filtration, washed with distilled water and acetone and dried at 80 °C overnight. Calcination was performed at 550 °C in air, with a 1 °C/minute ramp and a 6 hour dwell time at 550 °C. Powder X-ray diffraction experiments (using Cu Kα radiation, PANanalytical Empyrean in Bragg-Brentano mode at room temperature) was used to confirm phases and purity of samples.

S1.2 Isotopic Enrichment

The isotopic enrichment of the samples was performed in two different ways as described in the main text and in previous work. Method 1 involved the mixing of ~25 mg of a calcined zeolite with 25 μL of water enriched to 40% in ^{17}O in a Bruker 4 mm HRMAS PTFE insert, which was in turn sealed in a 4 mm rotor for acquisition of MAS NMR spectra at regular intervals, from immediately after mixing up to 212 days. In this "slurrying" method the sample is always in contact with water. The alternate method 2 involved mixing 50 mg of zeolite with 0.5 mL of water enriched to 40% in ^{17}O in a small sample vial and the mixture either stirred at 350 rpm using a magnetic stirrer or agitated using a shaker. After seven days contact with water the sample was filtered and dried at 80 °C overnight in an oven. The advantage of method 1 is that the same material can be studied over time (up to several months) but the ^{17}O NMR single-pulse spectra can be dominated by the water (although this does not appear in the MQMAS NMR spectra). The advantage of method 2 is that this dominant free water signal is removed by the drying step, allowing easier quantification of the enrichment. All stirred and shaken samples were exposed to the same drying regime (80 °C overnight) to ensure experiments were as comparable as possible.

S1.3 Solid-state NMR Spectroscopy

All ^{29}Si solid-state NMR measurements were performed using a Bruker Avance III spectrometer equipped with a 9.4 T wide-bore magnet, operating at a Larmor frequency of 79.47 MHz. All measurements were performed using a Bruker 4 mm HFXY probe at a spinning frequency of 10 kHz. ^{29}Si MAS NMR spectra were acquired using a recycle interval of 120 s, with a radiofrequency nutation rate of ~83.3 kHz and signal averaging of between 184 and 544 transients. $^{1}H/^{29}Si$ CP MAS NMR spectra were acquired using a recycle interval of 5 s using a ^{1}H $\pi/2$ pulse with a radiofrequency nutation rate of ~71.4 kHz and a 5 ms contact pulse (ramped for ^{1}H) for the transfer of magnetisation from ^{1}H to ^{29}Si, with signal averaging of 400 transients. During acquisition, TPPM15 ^{1}H heteronuclear decoupling was applied with a radiofrequency nutation rate of ~71.4 kHz. For the starting CHA samples the level of Q^3 silanols could be quantified (~3%) using single-pulse ^{29}Si NMR spectra but for AFI, FER and MFI there were no discernible Q^3 signals in the single-pulse or $^{1}H/^{29}Si$ CP MAS NMR spectra after the same data collection time (Fig. S1).

All ^{17}O solid-state NMR measurements (Figs. S2 and S7 and figures in the main text) were performed using a Bruker Avance III spectrometer equipped with a 14.1 T wide-bore magnet operating at a Larmor frequency of 81.34 MHz. A Bruker 3.2 mm HX probe was used with a spinning frequency of 20 kHz. ^{17}O MAS NMR spectra were acquired using a recycle interval of 1 s using a $\pi/12$ short flip angle pulse with a radiofrequency nutation rate of ~71.4 kHz and signal averaging between 128 and 5120 transients. ^{17}O triple-quantum MAS spectra were

acquired using a z-filtered pulse sequence, with a radiofrequency nutation rate of ~71.4 kHz for the triple-quantum excitation and conversion pulses, while the final CT-selective pulse had a nutation frequency of ~14.2 kHz. The recycle interval used was 1 s, with between 840 and 1200 transients acquired for each t_1 increment. The MQMAS spectra are shown after a shearing transformation to allow the projection of the isotropic spectrum directly in δ_1.

All ^{29}Si chemical shifts are shown in ppm relative to $Si(CH_3)_4$, using the $OSi(CH_3)_3$ resonance of octakis(trimethylsiloxy)silsequioxane (Q_8M_8) (δ = 11.5 ppm) as a secondary reference. All ^{17}O chemical shifts are shown in ppm relative to (natural abundance) H_2O (δ = 0 ppm).

Quantification of the level of ^{17}O enrichment was calculated using 40 % ^{17}O-enriched α-alumina as a reference standard. The integrated central-transition signal intensity in the short flip angle ^{17}O MAS NMR spectrum was determined for both α-alumina and the zeolite sample of interest, to ensure no differences in nutation rate as a result of different quadrupole couplings. This intensity was then scaled to account for any differences in the number of transients averaged and the mass of the materials. From a comparison of the carefully corrected intensities the number of moles of ^{17}O present in the sample, and therefore the absolute level of isotopic enrichment, can be estimated.

S1.4 Neutron Diffraction

The powdered samples of ^{nat}O CHA zeolite (i.e., a zeolite made from natural abundance oxygen) and ^{18}O-enriched CHA zeolite were prepared using the stirring method as described above, to match as closely as possible the samples used in the NMR experiments. The neutron diffraction experiments were performed at the Institut Laue-Langevin (Grenoble, France) using the D4c Disordered Materials Diffractometer which is optimized for high-accuracy PDF analysis experiments, including NDIS. The short neutron wavelength of 0.4992 Å allowed a high Q_{max} and therefore good r-space resolution in the final Radial Distribution Function (RDF), where $Q = (4\pi/\lambda)\sin\theta$, and 2θ is the diffraction angle. The samples were loaded into standard sealed vanadium cans of i.d. 6.68 mm and o.d. 6.98 mm and tapped to stabilize their heights within the cans. The height of the samples and the net sample masses were then measured to determine the sample powder packing fractions. Approximately 18 hours of data collection for each sample provided sufficiently high counting statistics. Additionally, background scans were performed for the empty containers, the empty instrument and a vanadium standard sample for diffraction intensity normalization, making use of the measured packing fractions for each sample. Data reduction and data analysis were performed following standard procedures, with the Correct programme[2] used to make the angle-dependent attenuation and multiple-scattering corrections along with the background subtraction and absolute intensity normalization to barns/str/atom. Standard checks against theoretical scattering intensities revealed that each sample contained roughly 6 atomic-% hydrogen as adsorbed water. This H content was then accounted for in the intensity normalization, such that only the estimated 0.8% difference in H content between the two samples had a small effect on the First Order Difference (FOD) and resulting RDF.

The sharpness of the peaks in Fig. 3a (main text), due to very well-defined distances in the zeolite crystalline structure, gives rise to some unavoidable Fourier “ripples” around each peak, and such Fourier artifacts are naturally exaggerated somewhat by the small diffraction signal used to calculate the FOD. Otherwise, the stronger, clearly robust peaks occur at all interatomic distances in the ^{nat}O (Fig. 3a black) and ^{18}O (Fig. 3a red) curves, but in the FOD (Fig. 3a gold curve) occur only at interatomic distances involving O sites where ^{16}O atoms have been replaced

by ^{18}O. Furthermore, the areas of the peaks in the FOD (Fig. 3a) are proportional to O-atom coordination number and the level of ^{18}O enrichment, since the latter affects the average coherent scattering length at the O-atom sites of the zeolite structure. The interatomic distances for the CHA zeolite structure shows that only O-O distances contribute to the peaks at 2.62 Å in all three curves. Since the intensity of these peaks depends on the average coherent scattering lengths, varying the enrichment level changes the normalised intensity of the peaks at 2.62 Å. When the correct enrichment has been found the three curves will have the same intensity. We found this to work best for an ^{18}O enrichment of 50%. Note that the overall amplitude of the FOD curve is sensitive to the level of ^{18}O enrichment, which allowed another self-consistency check; namely that 50% ^{18}O enrichment also scaled the FOD curve such that it dips to zero, within Fourier ripple noise, at 5.35 Å where there are no interatomic distances involving O atoms in the zeolite structure. One should remember that in the NMR experiments the water used in the exchange reaction is only enriched to 40% in ^{17}O. If we assume the level of enrichment will scale linearly with % enrichment of the water, this is equivalent to an enrichment in Si-O-Si of ~42% if 100% enriched water were to be used. The neutron diffraction enrichment (50%) is slightly more than this NMR-derived result (42%) but given the very small difference in neutron scattering between the oxygen isotopes this mismatch between NMR and neutron diffraction results is not unreasonable (see uncertainty estimates below).

Neutron diffraction refinement approach

All structural models were refined against the neutron PDF data by a real-space least-squares approach using TOPAS Academic V6. The DFT geometry-optimised structures was used as a starting model in a P1 space group setting of the CHA structure. Water molecules were treated as discrete rigid bodies, with 14 in total per unit cell. The simulated annealing approach integrated in the TOPAS program followed by subsequent least-squares refinement was carried out on the water positions and occupancies. The occupancies of all the water molecules were constrained to be equal. To be consistent with the PDF normalisation described above, oxygen occupancies of the oxygen sites were set to 50% ^{18}O. The final cycles of least-squares refinement included variation in the unit cell parameters, atomic positions and a damping factor, dQ. A SoperLorch function together with a function to model the correlated motion of atoms were used to fit the r-dependent broadening in the PDF data. The refinement range was 1.4 to 30 Å. The final weighted profile agreement factors (Rw %) and water occupancies for the four refinements carried out are shown in Table S1. Final atomic coordinates are given in the attached crystallographic information files (cifs). It should be noted that the structures reported are not necessarily the same as those that would result of refinement against Bragg diffraction data (e.g., Rietveld refinement against powder diffraction data). This is because the PDF(r) function is a histogram of interatomic distances (internuclear distances in the case of neutron diffraction). Therefore, the final refined structural model is just one possible arrangement of atoms that is consistent with this histogram of distances. For the CHA framework structure, the interatomic distances are well known and the refinements against PDF data reproduce these distances well. However, the location of water molecules inside the cages of the zeolite derived from the PDF refinements are not a unique set of atomic positions, merely one set that is consistent with the histogram of interatomic distances. However, the improvement in fits against the PDF data once these water molecules are included is very strong proof that there is scattering from within the pores of the zeolite.

Uncertainties in the neutron diffraction experiment
Neutron Diffraction with Isotope Substitution (NDIS) experiments require accurate normalisation of diffraction intensities to an absolute intensity scale for two chemically identical samples differing only in isotopic composition, after having taken into account sample attenuation corrections and the accurate subtraction of low and stable instrument backgrounds, in order to isolate the desired First-Order Difference (FOD) function containing information about the atomic environment of the substituted element in the sample's structure. This FOD as a Q-space function, as well as the total diffraction patterns for each of the two chemically identical samples (also Q-space functions), can then be Fourier transformed to produce their corresponding real-space Pair-Distribution Functions (PDF).

Since the Q-range of a neutron diffractometer is not infinite, nor is its Q-space resolution perfect, the Fourier transform to real-space results in the appearance of some "Fourier artifacts", generally in the form of "ripples" at low r-values, that can be identified e.g. by performing Fourier transforms with different Q-ranges: Those features in the PDF(r) that change significantly with Q-range are generally artifacts, while those that change little and in predictable ways are generally due to robust structural features in the sample. In addition, the Fourier artifacts of the two total diffraction patterns are generally quite similar, but could lead to large residual artifacts if the two PDF(r) functions were to be simply subtracted. This is why their subtraction is performed first in Q-space, being the space of the experimental measurement, in order to produce the FOD(Q) function, which is then Fourier transformed into r-space, thus cancelling out the bulk of the Q-space features that could contribute to large residual Fourier artifacts in the FOD-PDF of Figure 3(a).

Please also recall the well-known fact that it is not possible to calculate statistical error bars for the result of a Fourier transform. However, the uncertainties in the amplitudes of the plotted experimental RDF(r)/PDF(r) functions of Figures 3 (a), (b) and (c) can be estimated from the tests that we performed in identifying robust structural features of the PDF(r) functions as compared to Fourier artifacts, e.g. by adjusting the Q-range for the Fourier transform.

As concerns the uncertainty in the enrichment estimate of 50% as obtained from the FOD-PDF result, we described in the text that we found the adjustment of this single parameter of enrichment (which affects strongly the calculated normalization and also somewhat the shape of the FOD-PDF) to match the amplitude of the O-O peak at 2.62 Å to the experiment while simultaneously noting the amplitude at 5.35 Å is zero (where there are no O-containing atomic pair distances). Furthermore, a change in this enrichment by 5 % leads to these features clearly deviating from their expected values (i.e. they don’t match the experimental results at all well). We therefore conclude that we have determined the enrichment of the ^{18}O sample used in the neutron scattering experiment to be "50 % with an uncertainty of +/– 5 % ", where strictly speaking, one can take this "+/– 5 %" to be a fractional "standard deviation" that was determined empirically.

The final question is how this compares to the NMR experiments. A complication is the different enrichment levels available for water – 40% enriched for ^{17}O while 98% enriched for ^{18}O. The measured enrichment level for ^{17}O enriched is 17%, with an estimated uncertainty of around 1 to 2 % on this measurement. To scale this enrichment to be equivalent to that when using 98%

enrichment we need to multiply the 17% by 2.45, which gives an estimated equivalent NMRenrichment of ~42% with an estimated uncertainty of now at least ~+/– 3%. Therefore, the two measurements of 50% and 42% are broadly the same when uncertainties are taken into account, and we would certainly not be confident in saying that they are different. We perhaps would not expect them to be exactly the same as it is difficult when using different commercial reagents (such as the two types of enriched water) and then treating them in exactly the same manner. We believe that small differences in stirring (e.g. stirring rate) could easily account for the differences in enrichment levels measured. Again, it is not really the absolute enrichment levels that are the major point here, but that enrichment occurs at all.

S1.5 Computational Methodology

Molecular dynamics computational methodology

Chabazite models were constructed using the CHA topology (36 T site) model from the International Association (IZA) Structure Commission database (iza-online.org). The initial cell parameters were $a = b$ 13.67500 Å, c = 14.76700 Å, with angles $\alpha = \beta = 90°$ and $\gamma = 120°$. For all anhydrous models, including those with defects, the cells were held at the experimental size listed in the IZA database. For hydrated models, the unit cells were optimised.

Defective frameworks were constructed according to the Q^3 (axial) defect structure established in previous works on aluminosilicate zeolites (reference 20 in the main text). This structure assumes the hydrolysis of a single siloxane bond to form two Q^3 hydroxyls, followed by the inversion of one Si tetrahedron. Thus, two, colinear Q^3 silanol groups are produced per unit cell. Reactive neural network potentials (NNP) were employed using molecular dynamics simulations (NNP-MD, 1.05 ns total timescale with a timestep of 0.5 fs). A water loading of 14 molecules (14w) per unit cell was chosen to closely match the density of liquid water (1 g cm^{-3}). MD simulations of duration 1.05 ns were performed in the NVT ensemble at a temperature of 350 K, with a timestep of 0.5 ps, utilising a Nosé-Hoover thermostat for thermal control. The initial 50 ps was considered to be the equilibration phase and was discarded from subsequent statistical and structural analysis. The lowest energy configuration sampled from snapshots taken every 0.05 ps along the trajectory was extracted and subjected to local geometry optimisation, according to the BFGS algorithm as implemented in the atomic simulation environment. Forces were considered to be converged once they dropped below the threshold of 0.05 eV Å^{-1}.

The lowest energy structures from each MD trajectory were then optimised using both Density Functional Theory (DFT) and NNP, to calculate the energy of reaction of forming a defect at each site in the presence of a water cluster. DFT calculations were used to provide a comparison for the structures and energetics derived using the NNPs. Local re-optimization of NNP local minima was performed using the SCAN meta-GGA exchange correlation functional, augmented with the Grimme D3 dispersion correction with Becke-Johnson damping. Calculations were performed within the VASP 5.4 package. The basis set was expanded to a kinetic energy cutoff of 700 eV, and self-consistency was considered to be achieved with an energy difference of 1×10^{-4} eV. Gaussian smearing, with a width of 0.1 eV was employed to aid in electronic convergence. The Brillouin zone was sampled with a k-point grid of $1 \times 1 \times 1$, and local minima were defined by reaching convergence criteria of force less than 10^{-3} eV Å^{-1}.

MD-derived energetics

The reaction energy for the formation of the various Q^3 defects was calculated using the following equation:

$$E_r = E(Q^4.nH_2O) - E(Q^3.(n-1)H_2O)\ ,$$

where the relevant number of water molecules are encapsulated within the zeolite pore. For NNP-MD, the energies quoted are average energies extracted from the simulation. Fig. S8 shows the reaction energies for all three methods and both levels of water loadings. With the exception of O4 (14w), all reaction energies are endothermic. In general, NNP-MD agrees closely with that optimised using the NNPs, implying that the simulation primarily explores the low-lying regions of configuration space, close to the local minima. Results are shown in Fig. S9 and Table S2.

DFT calculations of NMR parameters

Calculations were carried out using the CASTEP FT code (version 24), employing the gauge-including projector augmented wave (GIPAW) approach to reconstruct the all-electron wavefunction in the presence of a magnetic field. Calculations were performed using the GGA PBE exchange-correlation functional, with a D3 semi-empirical dispersion correction with Becke-Johnson damping. Core-valence interactions were described by ultrasoft pseudopotentials, and scalar relativistic effects were accounted for using ZORA. A planewave energy cutoff of 60 Ry (~816 eV) was used, with the first Brillouin zone sampled through a Monkhorst-Pack grid with a reciprocal space grid spacing of 0.04 2π Å^{-1}. Calculated isotropic chemical shifts, δ_{iso}, were obtained from the corresponding the isotropic shielding, $\sigma_{iso} = (1/3)\mathrm{Tr}\{\sigma^{PAS}\}$, using a reference shielding of 255.4 ppm for ^{17}O and 329.5 ppm for ^{29}Si, determined by comparing experimental and computed values for SiO_2 cristobalite. Note that there is a known temperature dependence of the ^{29}Si isotropic chemical shift for SiO_2 cristobalite of ~0.0113 ppm / K, which has been taken into account, although this has been shown to be dependent on the defect level of the material studied. The ^{17}O quadrupolar coupling constant, $C_Q = eQV_{ZZ}/h$, where Q is the nuclear quadrupole moment, which was 25.58 mb for ^{17}O. The asymmetry parameter, $\eta_Q = (V_{XX} - V_{YY}) / V_{ZZ}$. Calculations were performed for an idealised CHA framework with a $Si_{36}O_{72}$ unit cell obtained from the IZA, which was geometry optimized prior to the calculation of the NMR parameters. No water molecules were added.

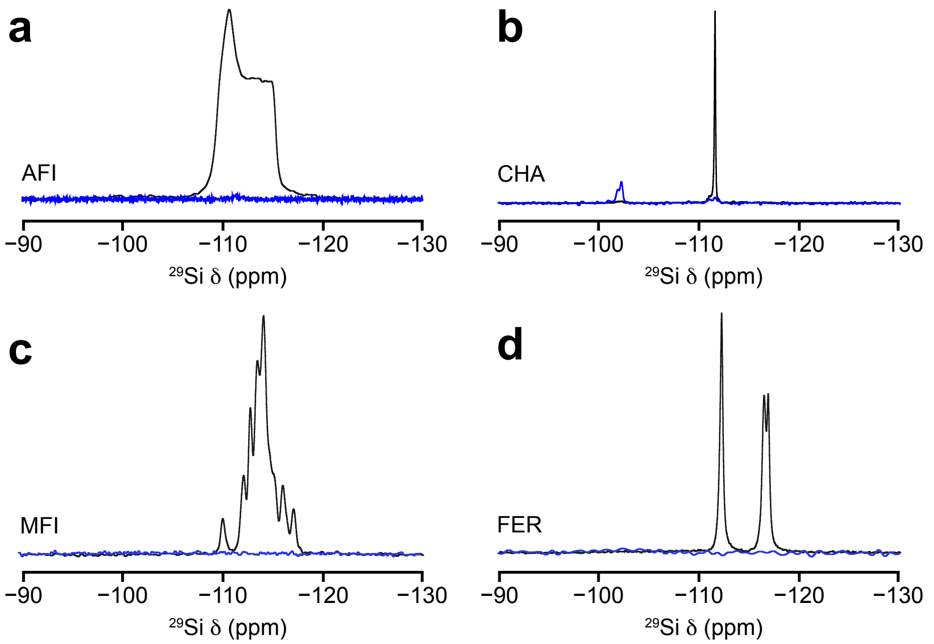

Fig. S1.

Single-pulse ^{29}Si MAS NMR spectra (black) and ^{1}H/^{29}Si cross polarisation (CP) MAS NMR spectra (blue) for (**a**) AFI, (**b**) CHA, (**c**) MFI and (**d**) FER. Note that the CP MAS NMR spectrum for CHA is the only one to show evidence of signal due to silanol defects (~3% Q^3 silicon species at δ = ~–102 ppm). Note that acquisition (9.4 T, 10 kHz) and processing parameters were the same for all these spectra.

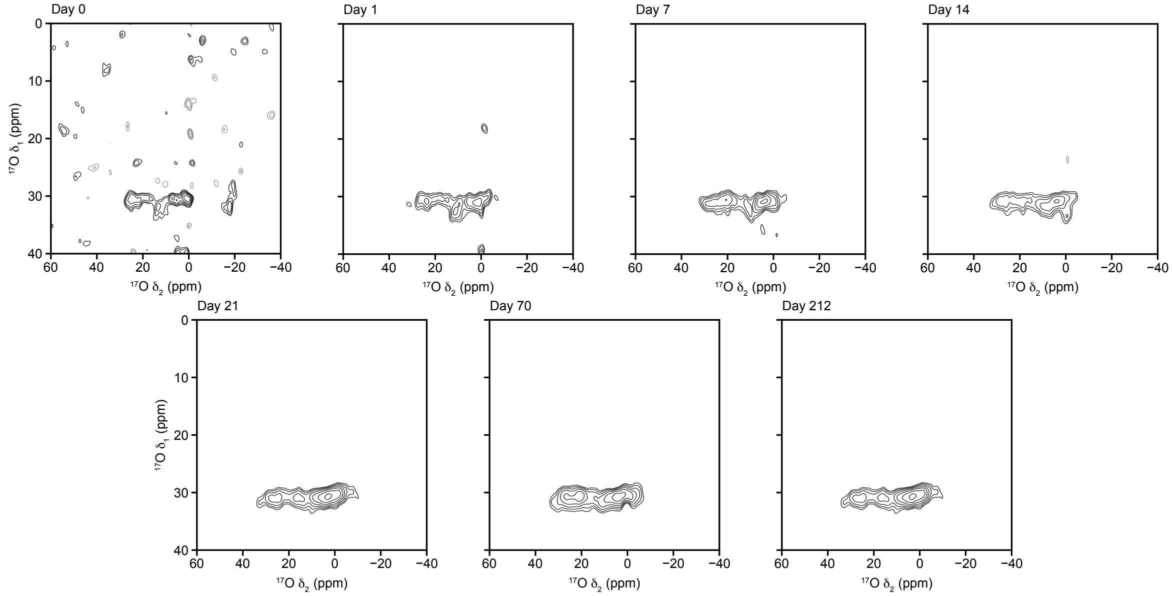

Fig. S2.
The evolution triple-quantum of ^{17}O (14.1 T, 20 kHz) MAS NMR spectra with time during which calcined zeolite AFI was slurried with 40% ^{17}O-enriched water.

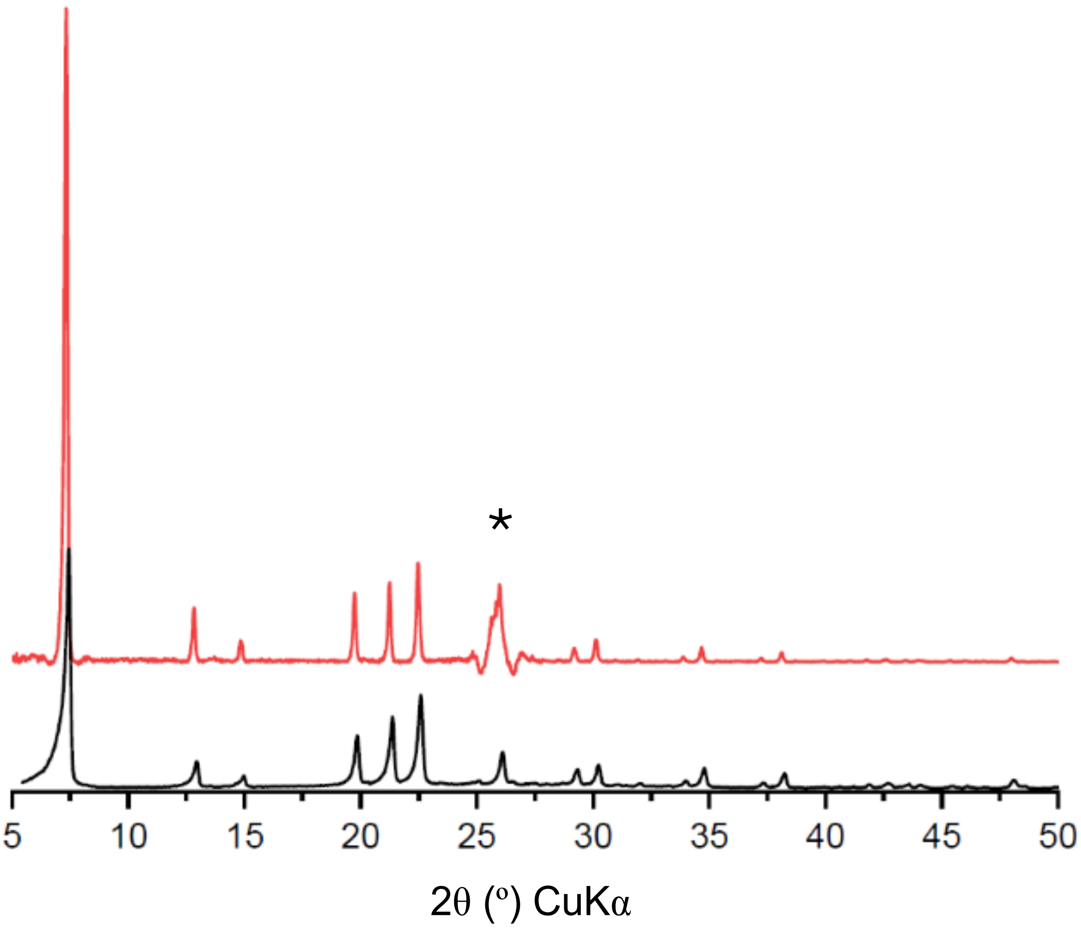

Fig. S3.
Comparison of PXRD patterns for calcined siliceous AFI (bottom) and ^{17}O-enriched siliceous AFI (top) after slurrying for 212 days. The asterisk marks a peak from the sample holder.

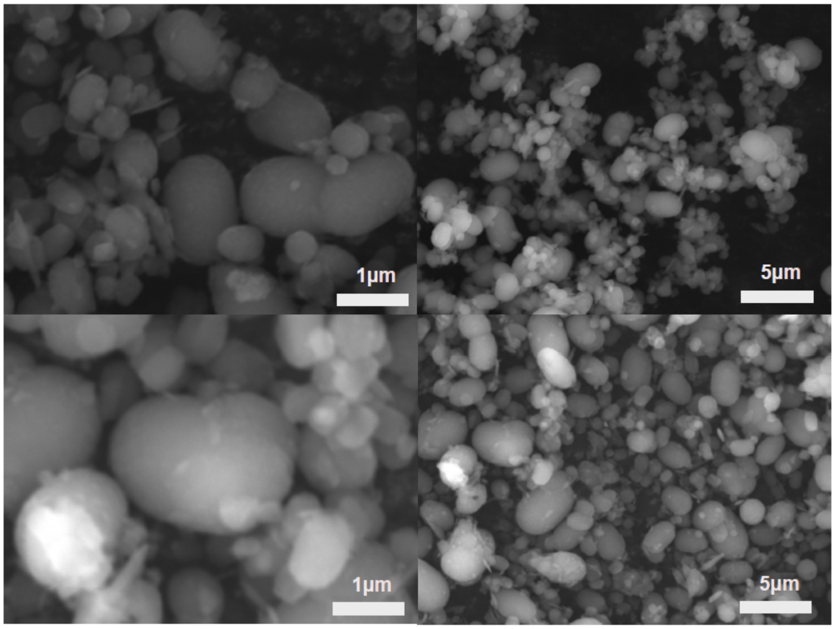

Fig. S4.
SEM images of calcined siliceous AFI (top) and ^{17}O-enriched calcined siliceous AFI (bottom). The siliceous AFI zeolite crystal shape and size does not change after ^{17}O enrichment using slurrying.

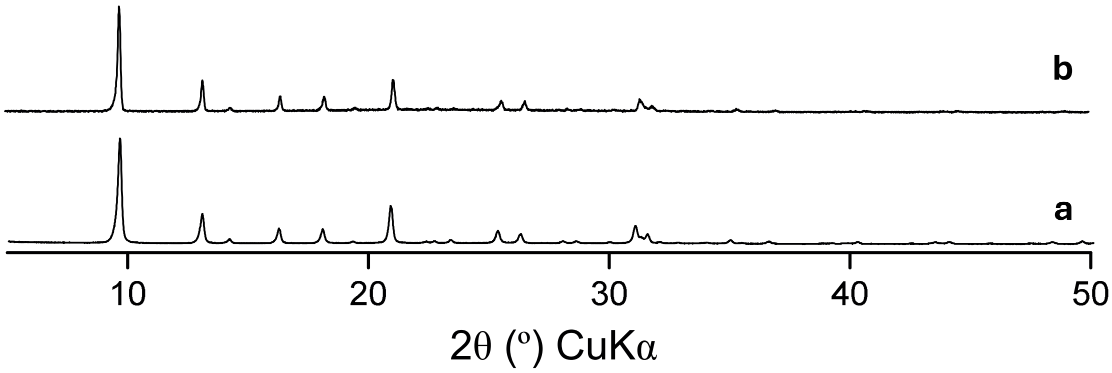

Fig. S5.
Comparison of PXRD patterns of siliceous CHA (**a**) before exposure to water and (**b**) after shaking in water for seven days.

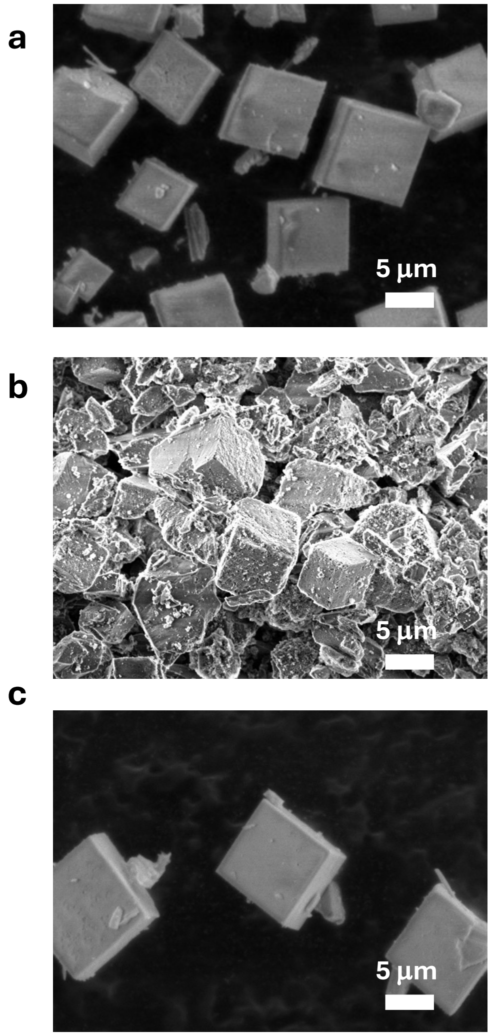

Fig. S6.
SEM images of pure-silica CHA (**a**) before exposure to liquid water, (**b**) after seven days stirring in ^{17}O-enriched water showing the damage to the crystals and (**c**) after seven days shaking in ^{17}O-enriched water showing much reduced surface damage.

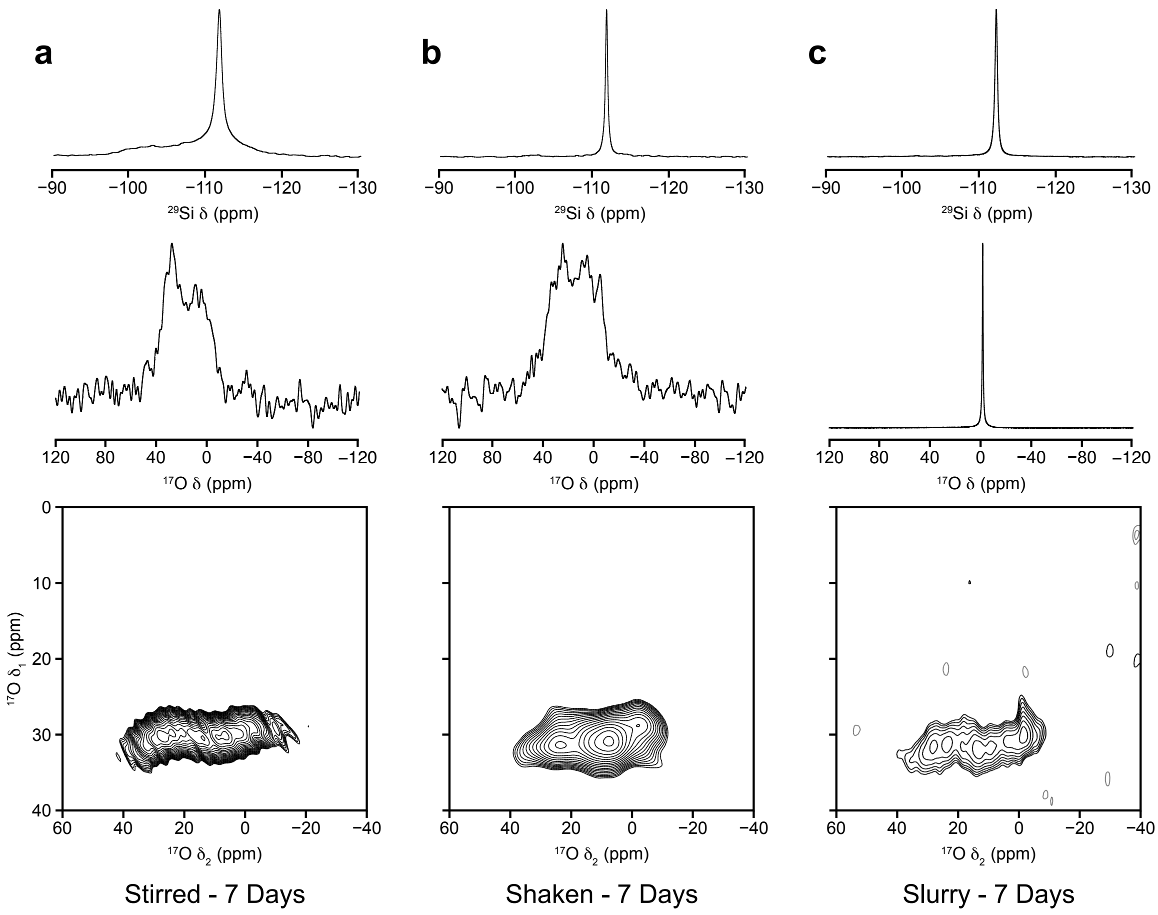

Fig. S7.
A comparison of ^{29}Si (9.4 T, 10 kHz) top, ^{17}O (14.1 T, 20 kHz) single-pulse (middle) and ^{17}O (14.1 T, 20 kHz) triple-quantum MAS NMR spectra of CHA samples isotopically enriched in different ways. (**a**) stirred for 7 days in 40% ^{17}O-enriched water, (**b**) shaken for 7 days in 40% ^{17}O-enriched water and (**c**) slurried for 7 days in 40% ^{17}O-enriched water.

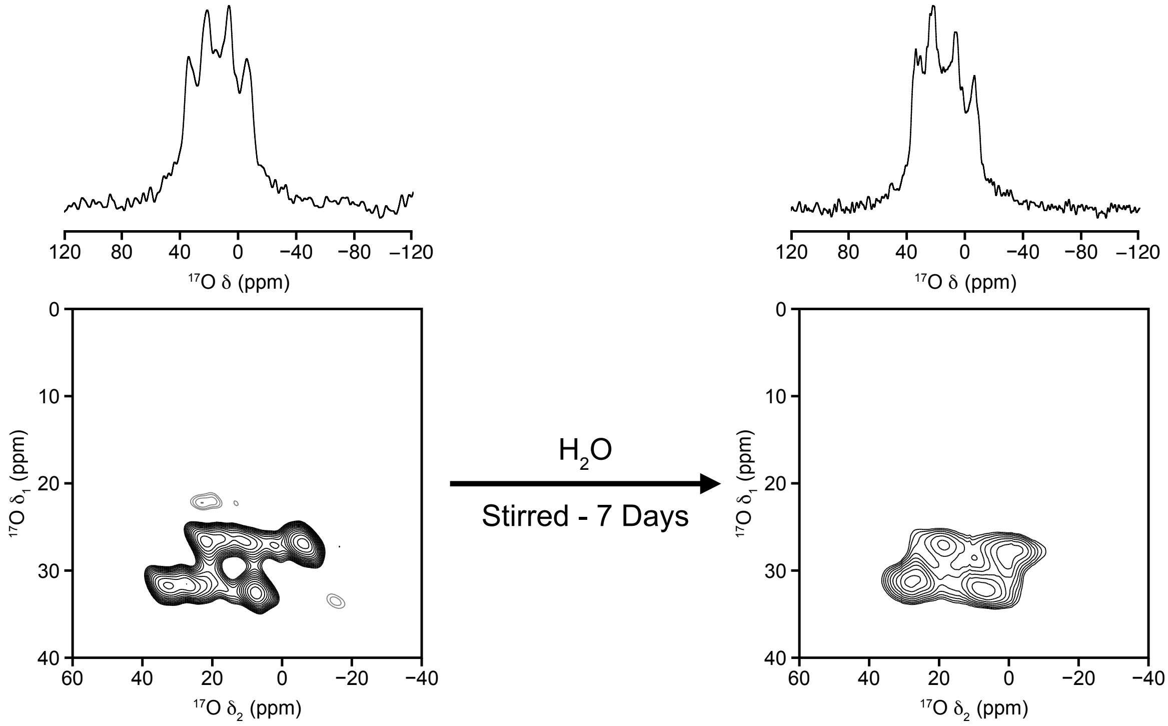

Fig. S8.

^{17}O (14.1 T, 20 kHz) single-pulse (top) and ^{17}O (14.1 T, 20 kHz) triple-quantum MAS NMR spectra (bottom) of CHA samples isotopically enriched with ^{17}O during synthesis before (left) and after (right) stirring in natural abundance water for seven days. This experiment tests for back exchange of ^{16}O for ^{17}O. The signals show a broadening in δ_1 but no conclusive evidence for selective exchange at any particular oxygen site in the framework.

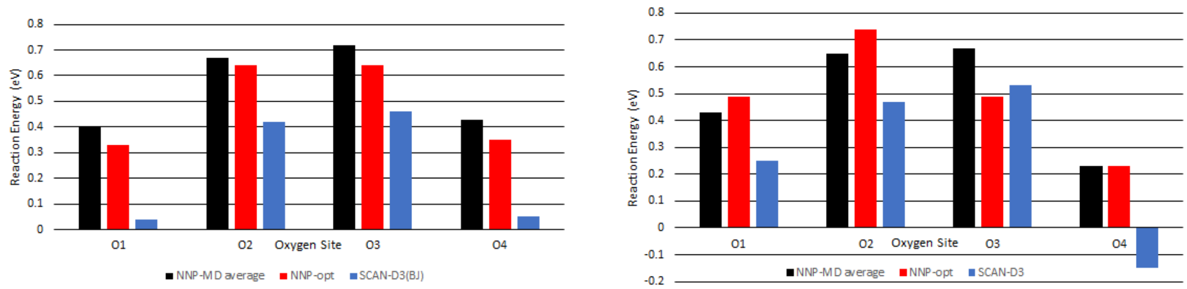

Fig. S9.
Calculated reaction energies for the defect formation reactions in anhydrous (left) and hydrated (right) Si-CHA, initiated at each of the four inequivalent oxygens (O1, O2, O3 or O4), for NNP-average energies, NNP-optimized energies and energies determined using SCAN-D3(BJ).

a

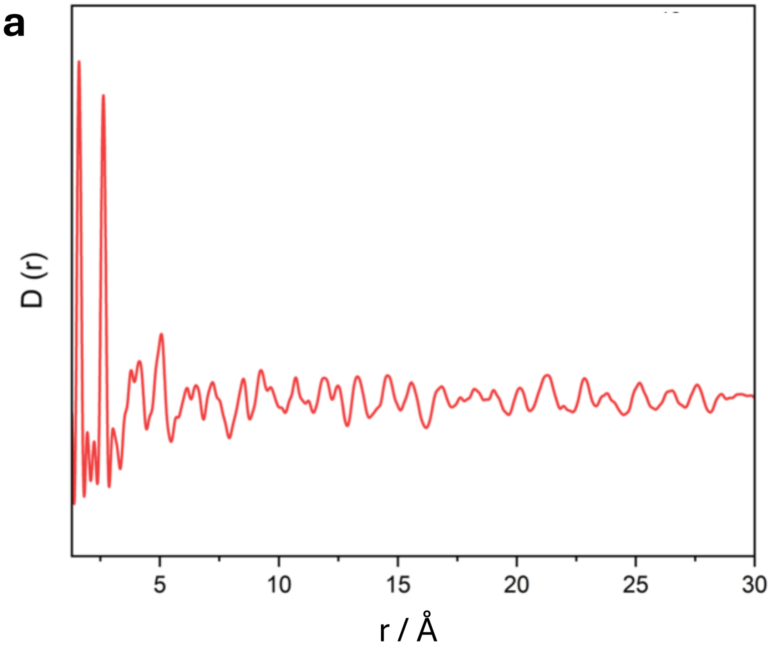

b

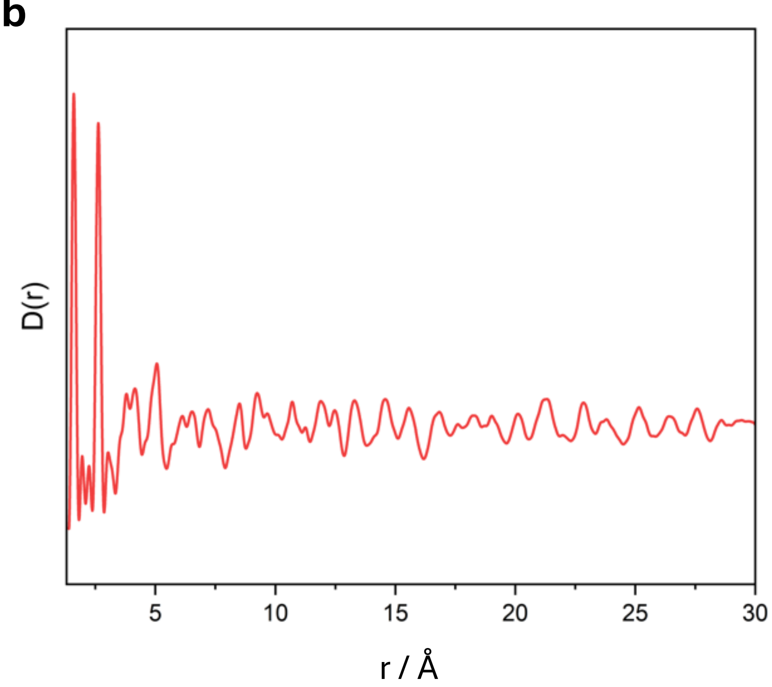

Fig. S10.
Neutron diffraction PDF data from 1.4 Å to 30 Å for (**a**) the unenriched CHA sample (after exposure to natural abundance H_2O for seven days) and (**b**) after treatment with 98% ^{18}O-enriched water for seven days.

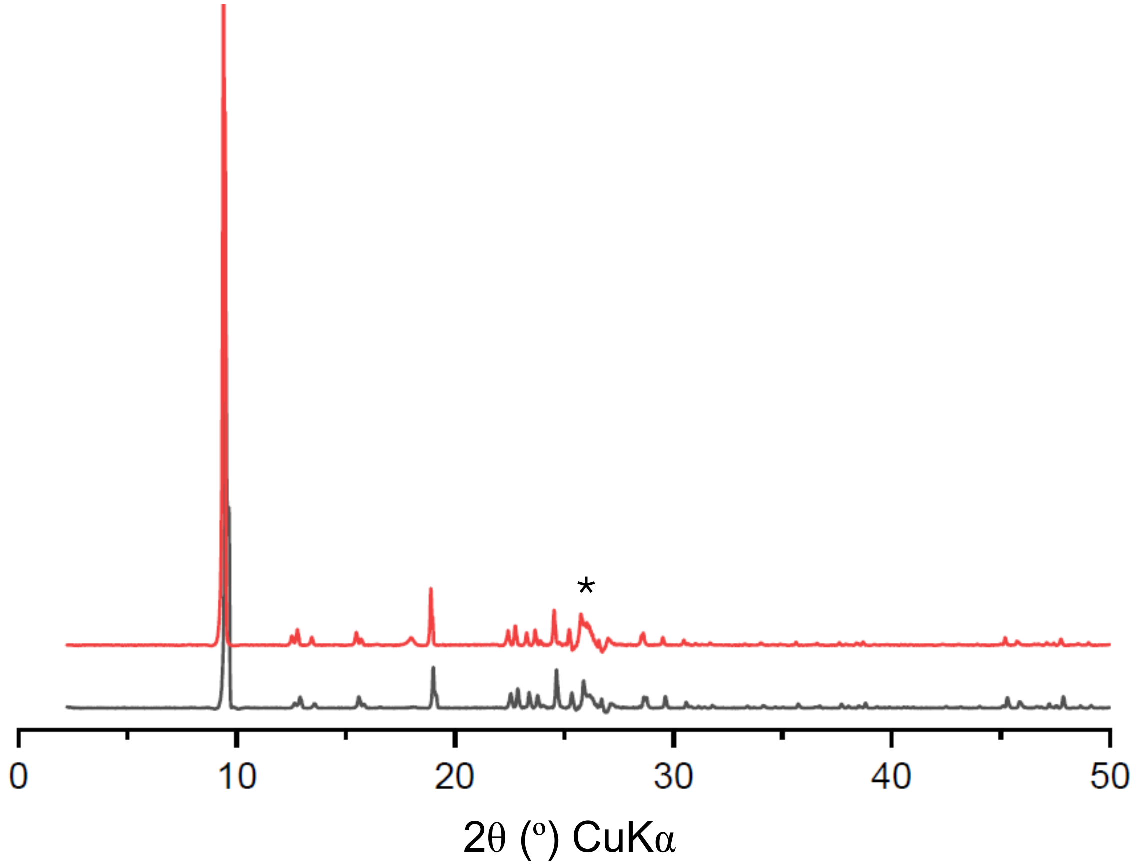

Fig. S11.
PXRD patterns of siliceous FER (bottom) before exposure to water and (top) after stirring in ^{17}O-enriched water for seven days. Note the extra reflection in the diffraction pattern at ~18° 2θ which indicates that a very small amount of breakdown of the FER has occurred. The peak from the sample holder is again visible at ~26 °2θ (marked by the *).

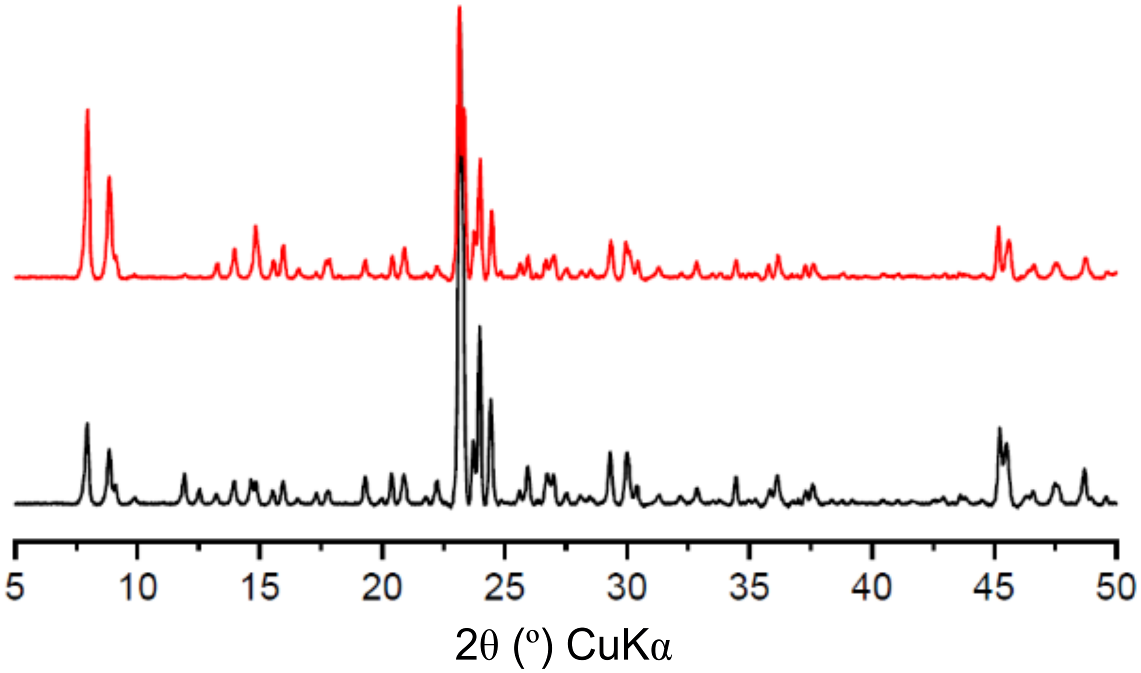

Fig. S12.

PXRD patterns of siliceous MFI (bottom) before exposure to water and (top) after stirring in 40% ^{17}O-enriched water for seven days. Note that all peaks in both patterns can be identified as MFI; differences in relative intensities between the two patterns are caused by differences in sample packing.

Table S1. Final profile agreement factors (Rw) and water occupancies (occ. %) for the PDF refinements for the unenriched (^{Nat}O) and enriched CHA samples with and without water in the pores.

**System**	**CIF name**	**Rw (%)**	**H_2O occ. %**
^{Nat}O without water	NatOCHA_without_Water.cif	20.16	N/A
^{Nat}O with water	NatOCHA_Water.cif	17.71	27.0
Enriched without water	EnrichedCHA_without_Water.cif	20.29	N/A
Enriched with water	EnrichedCHA_Water_Refined.cif	17.68	28.4

Table S2. Relative energetics (in eV) for silanol models of anhydrous and fully water-loaded CHA at NNP and DFT (SCAN-D3(BJ)) levels. The silanols are formed by breaking the Si-O-Si bonds at each crystallographically distinct oxygen site. Minimum energy sites for each water loading are indicated in bold. Water contents are in molecules per unit cell.

H_2O/u.c.	Silanol site	NNP (opt)	SCAN-D3(BJ)
0	O1	**0.00**	**0.00**
	O2	0.31	0.38
	O3	0.31	0.42
	O4	0.02	0.01
14	O1	0.26	0.41
	O2	0.51	0.62
	O3	0.27	0.68
	O4	**0.00**	**0.00**

Table S3. Calculated (using DFT) NMR parameters (isotropic chemical shift, δ_{iso}, quadrupolar coupling, C_Q and quadrupolar asymmetry, η_Q, predicted shift in the isotropic dimension of an MQMAS spectrum acquired at 14.1 T, δ_1) for an idealised, dehydrated SiO_2 CHA zeolite.

Species	σ_{iso} (ppm)	δ_{iso} (ppm)	C_Q / MHz	η_Q	δ_1 (ppm)
Si	443.0	–113.5[a]			
O1	214.0	41.4	–5.58	0.14	31.9
O2	228.6	26.8	–5.84	0.16	24.8
O3	226.4	28.9	–5.81	0.19	25.9
O4	216.6	38.8	–5.68	0.16	30.8

[a] Assuming a temperature dependence of –0.0113 ppm / K
